# Target of Rapamycin in Control of Autophagy: Puppet Master and Signal Integrator

**DOI:** 10.3390/ijms21218259

**Published:** 2020-11-04

**Authors:** Yosia Mugume, Zakayo Kazibwe, Diane C. Bassham

**Affiliations:** Department of Genetics, Development and Cell Biology, Iowa State University, Ames, IA 50011, USA; ymugume@iastate.edu (Y.M.); zkazibwe@iastate.edu (Z.K.)

**Keywords:** autophagy, plant growth, target of rapamycin (TOR), TORC1, TOR signaling

## Abstract

The target of rapamycin (TOR) is an evolutionarily-conserved serine/threonine kinase that senses and integrates signals from the environment to coordinate developmental and metabolic processes. TOR senses nutrients, hormones, metabolites, and stress signals to promote cell and organ growth when conditions are favorable. However, TOR is inhibited when conditions are unfavorable, promoting catabolic processes such as autophagy. Autophagy is a macromolecular degradation pathway by which cells degrade and recycle cytoplasmic materials. TOR negatively regulates autophagy through phosphorylation of ATG13, preventing activation of the autophagy-initiating ATG1-ATG13 kinase complex. Here we review TOR complex composition and function in photosynthetic and non-photosynthetic organisms. We also review recent developments in the identification of upstream TOR activators and downstream effectors of TOR. Finally, we discuss recent developments in our understanding of the regulation of autophagy by TOR in photosynthetic organisms.

## 1. Introduction

All living organisms require continuous monitoring and integration of signals from their environments for survival, growth, and homeostasis. The target of rapamycin (TOR) kinase complex, an evolutionarily-conserved serine/threonine protein kinase, senses and integrates environmental cues, such as nutrients and stress signals [1,2], and internal signals, like energy and hormones [3,4], to regulate growth and metabolism in both photosynthetic and non-photosynthetic organisms.

Our understanding of TOR signaling and cellular function began several decades ago when two TOR genes were first isolated from the budding yeast *Saccharomyces cerevisiae* [5,6]. Subsequently, TOR signaling, function, and structure have been elucidated in other non-photosynthetic organisms such as fungi, *Drosophila*, and mammals [1,7,8,9,10], and photosynthetic organisms including *Arabidopsis thaliana* and algae [11,12]. In fungi and metazoans, TOR forms two structurally and physiologically distinct complexes, termed TORC1 and TORC2 (TOR complex 1 and 2), which regulate cell growth and metabolism in space and time [6,13,14]. However, to date only TORC1 has been found in plants.

Cellular anabolic and catabolic processes must both be regulated to maintain homeostasis during growth. Given nutrient-replete or abundant energy conditions, TOR kinase is active and promotes anabolic processes leading to growth [3,15]. Conversely, TOR activity is inhibited in response to stress conditions or upon treatment with inhibitors such as rapamycin, leading to the activation of catabolic processes such as autophagy [16,17,18,19].

Autophagy is a stress-responsive process found throughout eukaryotes by which cell components, including macromolecules and malfunctioning or damaged organelles, are degraded and constituents recycled. Three functionally similar but mechanistically distinct forms of autophagy, including macroautophagy [20,21,22,23,24] and microautophagy [25,26,27,28] in animals, plants and yeast, and chaperone-mediated autophagy in animals [29,30], have been described. In this review, we focus on the most studied and well-characterized form, macroautophagy (hereafter referred to as autophagy). Autophagy is divided into distinct steps which are performed by autophagy-related (ATG) proteins, many of which have been well-characterized in a number of organisms [31,32,33,34]. These steps are themselves complex and include induction/membrane nucleation, elongation, fusion, cargo degradation, and recycling [22,35,36,37,38]. Autophagy proceeds by engulfment of the cytoplasmic components into double-membrane vesicles known as autophagosomes, which subsequently fuse with and release their content into the vacuole/lysosome lumen for degradation by hydrolytic enzymes [35,39]. The major rate limiting step in flux through the entire autophagy pathway (autophagic flux) is initiation/nucleation; however, the origin of the autophagosome membrane is debated and not well understood, although several reports suggest the endoplasmic reticulum as the major initiation site in mammals [40] and plants [41,42]. Autophagy is activated under a wide range of different conditions and in response to multiple signals. Although the basic mechanism and machinery for autophagy is the same in most cases, the upstream regulatory pathways may be distinct, depending on the specific input or intracellular/extracellular stimuli such as nutrient deficiency and stresses like hypoxia, energy, and oxidative stress [43]. The multistep nature of the autophagy process also allows multiple layers of regulation that can include modification of different components of the autophagy machinery functioning at different stages of the pathway.

Under normal nutrient-rich conditions, autophagy is maintained at low basal levels (basal autophagy) and its disruption may result in impaired homeostasis and abnormal conditions such as cancer and neurodegenerative disorders [44,45,46]. The low basal autophagy level is maintained by TOR-mediated phosphorylation of ATG13, preventing autophagy induction by the ATG1-ATG13 kinase complex [47,48,49,50]. However, stress conditions such as nutrient starvation and cellular energy deficit [19,51,52,53,54,55,56] trigger responses that reduce TOR activity, which in turn leads to autophagy induction. Although we focus on general non-selective autophagy, it is noteworthy that autophagy can be selective, and targeting of specific cell organelles and macromolecules to the lysosome/vacuole for degradation has been described in detail elsewhere [57,58,59,60]. This selectivity of autophagy adds yet another layer of regulation to achieve cellular homeostasis in different conditions. Whether TOR is involved in selective turnover of these organelles in most cases remains to be elucidated. Here, we discuss TOR complex structure and function and recent advances in our understanding of TOR signaling and control of autophagy, with a focus on photosynthetic organisms. We conclude by discussing possible future research directions.

## 2. TOR Complex Components

### 2.1. TORC1 Components

**Figure 1:**
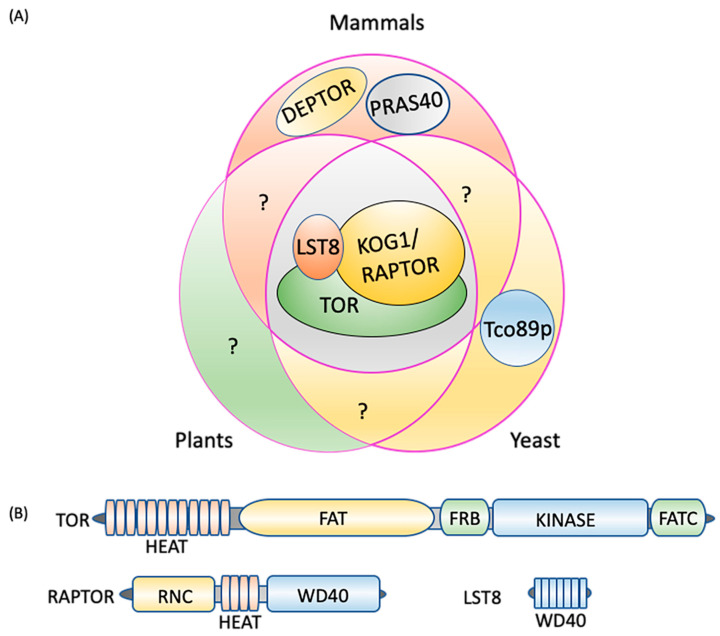
TORC1 composition. (**A**) Three core components, TOR, LST8, and RAPTOR/KOG1, are conserved in plants, mammals, and yeast. DEPTOR and PRAS40 are unique to mammals and Tco89p to yeast. (**B**) TORC1 protein domains. TOR consists of five distinct domains, the N-terminal HEAT repeats, FAT, FRB, kinase, and the C-terminal FATC. RAPTOR is composed of an N-terminal RNC domain, HEAT repeats in the middle section and a WD40 domain at the C-terminus, while LST8 is made up of seven WD40 domains. DEPTOR: DEP domain containing mTOR interacting protein; FAT: focal adhesion target/FRAP-ATM-TTRAP domain; FATC: FAT-carboxy terminal domain; FRB: FKBP12-rapamycin binding domain; HEAT: Huntingtin elongation factor 3-regulatory subunit A of PP2A-TOR1 repeats; KOG1: kontroller of growth 1; LST8: lethal with Sec13 protein 8; PRAS40: proline-rich Akt substrate of 40 kDa; RAPTOR: regulatory protein associated with TOR; RNC: RAPTOR N-terminus conserved domain; Tco89p: 89 kDa protein of TOR complex one; TOR: target of rapamycin; WD40: tryptophan-aspartic acid repeats of 40 amino acids.

In most eukaryotes, TOR assembles into two structurally and functionally distinct multiprotein, high molecular mass complexes known as TORC1 and TORC2 [6,14] (Figure 1A). To date, there is no evidence for the existence of TORC2-related proteins in plants or microalgae [2,61], suggesting evolutionary conservation of only TORC1. Additionally, the role of TORC2 in autophagy signaling is not well studied and therefore, we discuss only TORC1-related proteins and signaling in the subsequent sections. In yeast, plants and animals, TORC1 constitutes a catalytic subunit, TOR itself, a regulatory subunit, LST8 (lethal with sec thirteen 8), and RAPTOR (regulatory-associated protein of TOR/kontroller of growth 1 (KOG1) in yeast) [62,63,64]. Yeast-specific core components include Tco89p (89 kDa protein of TOR complex one), which is absent in mammals and plants [6,65], while the additional components of the mammalian TORC1 are DEPTOR (DEP-domain-containing mTOR-interacting protein), [66], and PRAS40 (40 kDa pro-Rich AKT substrate) [67] (Figure 1A). DEPTOR and PRAS40 have inhibitory activity toward mammalian TORC1 and regulate substrate recruitment and apoptosis in cancer cells [66,68,69,70]. Whereas these subunits appear to be absent in yeast and plants, it is possible that they are present but diverged in sequence and if this is the case, could they add yet another regulatory switch to TOR-autophagy signaling? For instance, these TORC1 components may activate autophagy by inhibiting TORC1 even in nutrient sufficient conditions in which autophagy is normally downregulated. Future research may uncover these or related components in yeast and plants and help decipher other autophagy regulatory mechanisms.

### 2.2. TORC1 Protein Domain Structures

The TOR protein kinase catalytic subunit consists of conserved N-terminal HEAT (Huntington-EF3-PP2A-TOR1) repeats (Figure 1B), which interact with RAPTOR and are required for substrate recruitment and membrane association. In mammals, the localization of TORC1 to the lysosome membrane is necessary for its negative regulation of autophagy when nutrients, particularly amino acids, are abundant [63,71,72] (see Section 3 for details). By contrast, the yeast TORC1 appears to constitutively localize to the vacuole membrane [73] whereas subcellular localization of plant TORC1 under different conditions needs further research. In the central region of TOR lies a focal adhesion target (FAT) domain potentially involved in interactions with partner proteins [64,74] and which contributes to kinase activation, a necessary step for TORC1 signaling. The catalytic Ser/Thr kinase domain is sandwiched between the FKBP12/rapamycin binding (FRB) domain, and the FATC (FAT C-terminus) domain at the extreme C-terminus. The FRB domain is the binding site for the FKBP12-rapamycin complex [5,6,61,75,76], while the FATC domain serves a scaffolding and kinase activation role [77].

RAPTOR is made up of a RAPTOR N-terminal conserved (RNC) domain, a HEAT domain, and a WD40 (tryptophan-aspartic acid repeats of 40 amino acids) domain at the C-terminus [78,79] (Figure 1B). RAPTOR binds to the HEAT repeat of TOR, selectively presents substrates to the kinase domain, and facilitates substrate phosphorylation [2,80,81,82,83]. RAPTOR also has a role in assembly and stabilization of the complex in mammals [78,79], and the conserved nature of the RNC and HEAT repeats in RAPTOR orthologs suggests a conserved role in other organisms. In *Arabidopsis*, knock-out of *RAPTOR* activates autophagy under non-stressed conditions, suggesting that RAPTOR plays a role in TOR-mediated autophagy repression [19]. LST8 is mainly composed of WD40 repeats, similar to the β-propeller fold of WD-repeat proteins [61,84] (Figure 1B). By interacting with the C-terminal domain of the catalytic subunit, LST8 contributes to TOR complex stability and catalytic activity regulation in yeast, plants and animals [85,86,87,88].

The existence of TORC1 as a multiprotein complex suggests different possibilities/avenues of signal integration and control. Given that cells experience constant fluctuations in the internal and external environment, it is plausible that a key protein such as TORC1 must adapt quickly and rapidly to these changes to maintain cellular function, and this can be achieved through regulation of different components. Moreover, could these subunits be involved in sensing of upstream signals, or could they mediate TOR interaction with other proteins to regulate autophagy? How does RAPTOR select the substrates to be presented to TOR? Could it have substrate-specific interacting motifs? Or is the structure modified for substrate binding? Future research may aid in answering these or similar questions to fully understand the cooperative processes by which TOR and associated proteins integrate different signals to regulate autophagy. [61] For instance, insight could be generated from structural studies of TORC1 using cryo- electron microscopy (Cryo-EM), for example; although a detailed structure has been generated for mammalian TORC1, more information is needed for plant TORC1 to provide a mechanistic understanding of TOR-autophagy signaling.

## 3. TOR Signaling in Non-Photosynthetic Organisms

TOR mediates a wide range of processes by integrating multiple upstream signals. In this section, we briefly discuss our current understanding of the TOR signaling network in non-photosynthetic organisms, with emphasis on regulation of autophagy.

### 3.1. Upstream Regulators of TORC1

**Figure 2:**
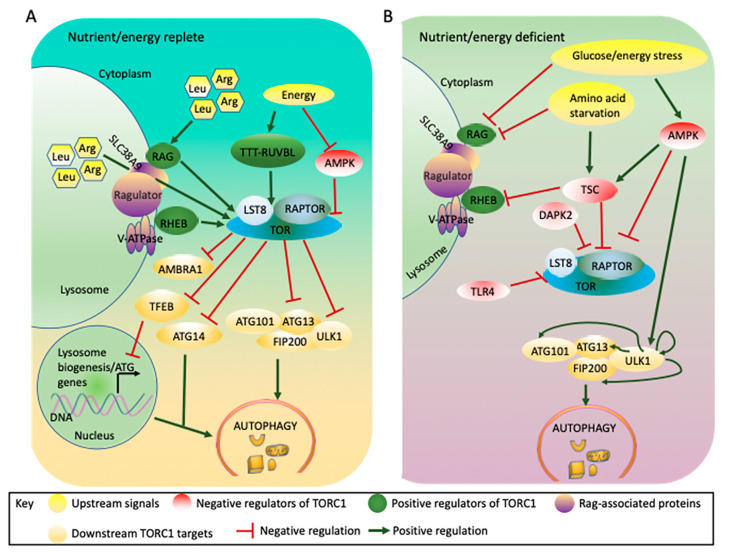
Mammalian TORC1 integrates signals from nutrients and energy to regulate autophagy. (**A**). TORC1 is activated by abundant nutrients/energy and phosphorylates components of the autophagy machinery including ULK1, ATG13, ATG14, AMBRA1, and TFEB, blocking autophagy induction. (**B**). Under nutrient/energy-deficient conditions, TORC1 is inactivated through either TSC or AMPK. Under these conditions, TSC associates with the lysosome and prevents the activation of TORC1 by RHEB, while AMPK phosphorylates RAPTOR and TSC leading to inactivation of TORC1. The loss of TORC1 activity prevents the TORC1-mediated inhibitory phosphorylation of ULK1 and ATG13, while autophosphorylation or AMPK-mediated phosphorylation of ULK1 leads to autophagy induction. Other factors such as DAPK2 phosphorylate RAPTOR under energy stress conditions, leading to autophagy induction, while the TTT-RUVBL complex facilitates TORC1 activation under energy- replete conditions, blocking autophagy induction. AMBRA1: Autophagy/beclin-1 regulator 1; AMPK: adenosine monophosphate activated protein kinase; Arg: Arginine; ATG: Autophagy related protein; DAPK2: Death-associated protein kinase 2; FIP200: focal adhesion kinase family interacting protein of 200 kDa; Leu: leucine; LST8: lethal with sec thirteen 8; RAG: Ras-related GTP binding protein; RAPTOR: regulatory associated protein of TOR; RHEB: Ras homolog enriched in brain; SLC38A9: solute carrier family 38; TFEB: transcription factor EB; TLR4: toll-like receptor 4; TOR: target of rapamycin; TSC: tuberous sclerosis complex; TTT-RUVBL: Tel2-Tti1-Tti2-RuvB-like AAA ATPase and ATP-dependent DNA helicase complex; ULK1: UNC-51-like kinase 1; V-ATPase: vacuolar-type H+-ATPase.

The known upstream stimuli that control TOR activity include nutrients, growth factors, and energy, which in mammals converge on the tuberous sclerosis complex (TSC), a negative regulator of TORC1 kinase activity [1,89,90,91] (Figure 2). However, TSC-independent TORC1 regulation via AMPK/SNF-1 (adenosine monophosphate-activated protein kinase in mammals/sucrose non-fermenting 1 in yeast) [92] or by amino acids via RAG (ras-related) guanosine triphosphatase (GTPase) has also been reported [71,72,93,94]. In mammalian cells, the upstream amino acids signal through specific sensors such as GATOR2 (GAP activity towards Rags 2) which activate TOR when amino acids are abundant. In both yeast and mammals, the inactivation of TORC1 induces autophagy, even in nutrient-replete conditions, indicating that TOR negatively regulates autophagy [95,96,97].

The lysosome plays a major role in growth factor- or amino acid-mediated TORC1 activation through RAG and RHEB (Ras homolog enriched in brain) GTPases [98,99,100], although RAG-independent TORC1 activators have also been reported [101,102] (Figure 2A). Moreover, mammalian SLC38A9 (solute carrier family 38) mediates the transport of many essential amino acids, such as leucine and arginine, out of the lysosome, resulting in activation of TORC1 and blocking autophagy induction [103,104,105]. Amino acid starvation abolishes the activation and recruitment of TORC1 to the lysosomal surface through RAG; TORC1 is therefore not recruited to its activator RHEB [106], potentially leading to autophagy induction [107].

When the ratio of cellular AMP to ATP increases in situations such as hypoxia [108,109], AMPK, an energy sensor, is activated and suppresses TORC1 signaling both through TSC and through direct phosphorylation of RAPTOR [92,110] (Figure 2B). In mammals, AMPK binds to the serine/threonine UNC-51-like kinase 1 (ULK1), a homologue of yeast ATG1, and simultaneously binds to and phosphorylates RAPTOR, resulting in inactivation of TORC1 and autophagy induction [52,92]. In addition, AMPK phosphorylates and activates TSC, which negatively regulates TORC1 activity during energy stress [93,94,111,112]. Glucose limitation also inhibits TORC1 in cells lacking AMPK through inhibition of the RAG GTPases, suggesting that more than one mechanism is involved in glucose sensing by TORC1 [113] and, therefore, in autophagy regulation.

Although several upstream TORC1 regulators have been discovered, it is possible that multiple activators or repressors of TORC1 exist to achieve homeostasis and regulation of autophagy in different conditions. For instance, while much attention has focused on a subset of amino acids such as arginine, leucine and methionine, whether a universal sensor of amino acids or other signaling molecules like nucleotides [114] exists remains to be determined. These upstream regulators may function in a cell- or tissue-specific manner to achieve a localized autophagy response [115]. In the future, it will be necessary to engage several methods, for example targeted phosphoproteomics and single cell RNA-sequencing, to facilitate the discovery of possible TORC1 interactors and components in TOR signaling in the face of different stimuli.

### 3.2. Downstream Effectors of TORC1

In both yeast and mammals, TOR phosphorylates ATG13-ATG1/ULK1 complex components to regulate autophagy, although the autophagy pathway downstream of TOR in both organisms is somewhat different. In yeast, under nutrient-rich conditions, TOR hyperphosphorylates ATG13, thereby preventing the formation of the ATG1-ATG13-ATG17 complex and blocking autophagy induction. However, nutrient starvation inactivates TOR and promotes the formation of this complex by rapid dephosphorylation of ATG13, resulting in autophagy induction [116,117,118,119].

Among multicellular organisms, TOR-mediated regulation of autophagy is best studied in mammals and *Drosophila*. The autophagy initiation complex in these organisms is composed of ULK1, ATG13, FIP200 (focal adhesion kinase family interacting protein of 200 kDa, a homolog of yeast ATG17), and ATG101 (an ATG13-binding protein) [120,121] (Figure 2). Unlike in yeast, this complex in mammals is formed even under nutrient-replete conditions [122,123], and the mechanism by which TOR-mediated phosphorylation of ATG13 regulates autophagy initiation is unclear. Apparently, under energy and nutrient replete conditions, TORC1 phosphorylates ULK1 and ATG13, blocking autophagy induction [52,124,125]. However, stress- or starvation-induced inactivation of TORC1 dissociates it from ULK1 and the phosphorylation of ATG13/ULK1 at inhibitory sites is mitigated by phosphatases [126,127,128]. At the same time, ULK1 is activated by AMPK-mediated or self-phosphorylation [124,129], and it phosphorylates ATG13, ATG101, and FIP200, leading to autophagy induction [123,130].

Other downstream effectors of TOR (Figure 2) that regulate autophagy in mammals include the transcription factor EB (TFEB), which controls the transcript levels of lysosomal biogenesis and autophagy genes [131,132,133]. Through the phosphorylation and inhibition of TFEB, mammalian TORC1 indirectly regulates autophagy [133,134,135,136]. TOR has also been implicated in phosphorylation of other ATG proteins, such as Atg14L [137,138] (in yeast and mammals) and AMBRA1 (Autophagy/beclin-1 regulator 1) [139] (in mammals), to regulate the early stages of autophagy induction [140].

Together, these studies in non-photosynthetic organisms indicate that cellular energy status and nutrients, particularly amino acids, determine the activation status of TORC1 and AMPK, which regulate autophagy through phosphorylation of the ATG13-ATG1/ULK kinase complex and other proteins. Although it is generally accepted that the role of autophagy is degradation and recycling of cell components for survival, rather than death, it is becoming clear that under certain conditions or in specific cell types, autophagy may accelerate cell death if it proceeds uncontrolled. It is, therefore, not surprising that through TOR, numerous brakes are engaged to ensure that autophagy is induced only when it is absolutely necessary. Moreover, TORC1-mediated autophagy regulation ensures that cells achieve a balance between degradation and biogenesis of cellular components, especially as TOR is also involved in several anabolic pathways. Future work will expand our understanding of the complex TORC1 signaling network and how it integrates signals from energy and intracellular nutrients to regulate autophagy.

## 4. TOR Signaling in Photosynthetic Organisms

As in non-photosynthetic eukaryotes, TOR is involved in a multitude of processes in plants, including plant organ initiation, growth and patterning, translation, transcription, cell cycle control, and autophagy [75,139,140,141,142,143,144,145,146,147,148]. In the following sections, we discuss upstream and downstream TOR-autophagy signaling network components in photosynthetic organisms, including algae and land plants.

**Figure 3:**
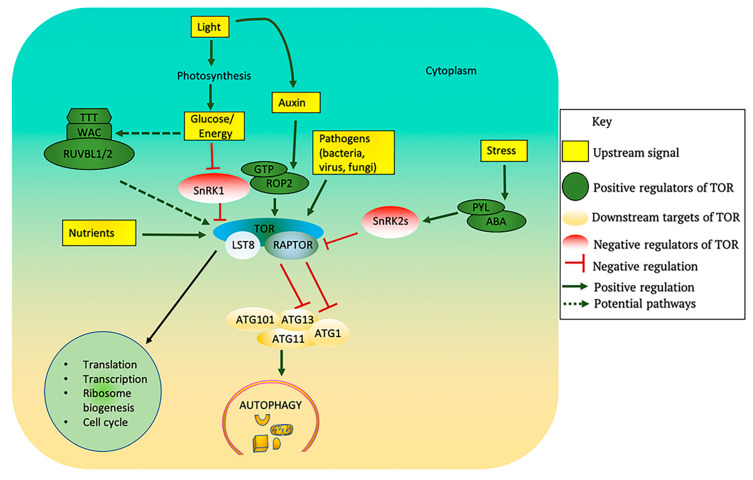
Upstream regulators and downstream processes of TOR in plants. TOR kinase activity is modulated by several upstream signals. Light promotes photosynthesis, which generates glucose that can inhibit SnRK1 and activate TOR. The *Arabidopsis* genome contains putative orthologous genes encoding TTT and RUVBLs, which may cause TORC1 dimerization and activation in response to glucose/energy availability. Light also promotes auxin biosynthesis which activates TOR via the small GTPase ROP2. Nutrients such as sulfur, nitrogen and phosphorus, and also amino acids, all activate TOR. Biotic stress due to some viruses, bacteria or fungi can also activate TOR. The plant stress hormone ABA triggers PYL-mediated activation of SnRK2s in response to stress and inhibits the TOR complex. Activation of TOR promotes the downstream processes of translation, transcription, ribosome biogenesis, and cell cycle while also blocking autophagy. Activated TOR phosphorylates ATG13 thus preventing its association with ATG1 and blocking autophagy activation. ABA: abscisic acid; ATG: Autophagy-related; LST8: lethal with sec thirteen 8; PYL: Pyrabactin Resistance 1 Like; RAPTOR: regulatory associated protein of TOR; ROP: Rho-like GTPases; RUVBL: RuvB-like AAA ATPase; WAC: WW domain containing adaptor with coiled-coil; SnRK1/2: SNF1-related protein kinase-1/2; TOR: target of rapamycin.

At least five major inputs, glucose/energy, hormones, nutrients, amino acids, and stress (biotic and abiotic), regulate TOR signaling in animals, yeast and in photosynthetic eukaryotes [1,2,149,150,151] (Figure 3). TOR integrates these upstream signals to control downstream processes which include autophagy (discussed below), transcription [17,152,153], translation [87,148,154], and cell cycle control [152]. In photosynthetic eukaryotes, the molecular and cellular mechanisms by which TOR integrates and coordinates signals from these inputs to activate downstream effectors are emerging.

### 4.1. Energy/Glucose and Light

Glucose (glc) generated from photosynthesis positively regulates TOR signaling in plants [152,155,156] and in turn activates root [152] and shoot [155,156] meristems. For shoot meristem activation, both light and glucose signals must be present to activate TOR, whereas in root meristems, glucose alone is sufficient. Thus, plants need to integrate light and glucose signals to activate TOR kinase for shoot development [156]. Light is important for promotion of auxin biosynthesis, and exogenous addition of auxin and glucose is sufficient to activate TOR, thus showing that auxin acts downstream of light [156]. Activated TOR kinase promotes activation of cell cycle genes by the E2Fa,b transcription factors to enable shoot and root meristem development [152,156].

The glc-TOR-E2Fa signaling pathway has also been implicated in thermotolerance and thermomemory [157,158]. Glc-activated TOR phosphorylates E2Fa which binds to the promoter of HLP1 (Hikeshi Like Protein 1), an ortholog of human Hikeshi. HLP1 binds to the promoters of many heat response genes and likely activates their expression and thus increases thermotolerance [157]. Additionally, TOR works in concert with p300 histone acetyltransferase 1 (HAC1) and HLP1 to modulate the chromatin acetylation of heat stress loci and impart short-term acquired thermotolerance. Glc-TOR signaling promotes long-term acquired thermotolerance by modifying the chromatin landscape at thermomemory-related loci by promoting H3K4 trimethylation (H3K4me3). Together, these studies show that glc-TOR signaling is important for control of thermotolerance and thermomemory, thus protecting plants from heat stress [157,158]. Recently it has been shown that autophagy negatively regulates thermomemory by selectively targeting heat shock proteins (HSPs) for degradation, and remains highly induced during thermorecovery [159,160]. It is therefore possible that glc-TOR signaling may also promote thermomemory by repressing autophagy and thus allow accumulation of heat shock proteins.

The details of the underlying molecular mechanisms through which glucose activates TOR in plants are still unclear. However, in mammals and flies the TEL2–TTI1–TTI2 (TTT)–RUVBL1/2 complex causes TORC1 dimerization, which is necessary for its translocation to the lysosomal membrane and activation in response to glucose/energy availability [161,162,163]. Since the *Arabidopsis* genome contains putative orthologous genes encoding TEL2–TTI1–TTI2 and RUVBLs [164], a similar mechanism may exist in plants, although this awaits experimental evidence. Glucose activation of TOR could also be mediated by inactivation of SnRK1, which is an ortholog of mammalian AMPK and is a major energy sensor [163,165]. Since SnRK1 is upstream of TOR [166], it may inhibit TOR activity by direct interaction and phosphorylation of RAPTOR1B [166]. However, under energy/glucose starvation conditions the primary target substrates of TOR and SnRK1 are not fully antagonistic [150,166]. Therefore, more studies are needed to determine whether TOR and SnRK1 target different phosphorylation substrates in response to different nutrient levels.

### 4.2. Phytohormones

In mammals, the TOR pathway responds to growth factors such as insulin to promote cell proliferation, growth, differentiation, and fate [3,167,168]. In plants, phytohormones such as auxins, jasmonic acid, abscisic acid, salicylic acid, brassinosteroids, and cytokinin have been implicated in the TOR signaling pathway [53,169,170,171]. Recently, it has been shown that cells induce autophagy for the purpose of temporary reprograming via large-scale degradation of cell components to remove the old program prior to activation of the new. This occurs in response to various hormonal signals [172]. It is not clear whether the autophagy induced during reprogramming is under the control of TOR in plants, although cellular dedifferentiation followed by reactivation of cell proliferation in animals after wounding is controlled by the level of TOR activity, providing a precedent for a role for TOR also in plant cell reprogramming [173].

Auxin controls multiple aspects of growth and development, including the cell cycle, cell division, elongation, and differentiation [174]. Auxin was first observed to activate TOR kinase in maize [175] and in *Arabidopsis* suspension culture [154,176] based on increased phosphorylation of T449 in S6K1. S6K1 is a direct TOR substrate [177] and a conserved indicator of endogenous TOR kinase activity [177]. As discussed above, light-induced auxin biosynthesis in combination with glc activates TOR and promotes growth of shoot meristems [156]. In mammalian cells the small GTPases RHEB and RAGs act as upstream activators of TOR in response to nutrients, growth factors, and amino acid signals [178,179,180]. Plant genomes, however, lack orthologs of the RHEB or RAG GTPases but instead contain members of a family of 11 ROP (Rho-like GTPases from plants) small GTPases [181,182], including ROP2, which interacts with and activates TOR kinase in response to auxin [183]. It is still not known whether other ROPs are involved in TOR signaling, although ROP4 AND ROP6 have been shown by yeast two-hybrid assay to specifically interact with TOR [183]. Consistent with its role in activation of TOR, exogenous addition of auxin to seedlings under various abiotic stresses blocked autophagy activation [19]. From these studies, it is likely that auxin regulates plant development partly via the TOR signaling pathway.

Auxin has also been implicated in activating the differentiation of xylem cells such as tracheary elements (TEs) [184]. This provides an intriguing connection between auxin and autophagy, as autophagy is induced in developing xylem cells and is required for tracheary element differentiation [185,186]. It is not clear what causes autophagy induction in differentiating TE cells; however, based on the role of auxin, an activator of TOR, and on the need for continued cell growth and differentiation during this developmental process, it seems likely that TOR activity would be maintained in the differentiating cells. This would mean that autophagy is activated despite the presence of active TOR, similar to the situation, for example, during ER stress activation of autophagy. This could be tested by assessing autophagy in TOR-deficient plants and by measuring TOR activity during xylem cell differentiation [19].

Unlike auxin, the plant stress hormone abscisic acid (ABA) inhibits TOR signaling [51]. Under non- stress conditions, TOR kinase phosphorylates the PYL (pyrabactin resistance1/PYR1-like) ABA receptors at a conserved serine residue. This phosphorylation negatively regulates PYL activity to inhibit ABA binding and interaction with downstream Protein Phosphatase 2C (PP2C) enzymes, preventing activation of stress responses. Under stress conditions, ABA triggers PYL-mediated activation of SnRK2, which phosphorylates the TOR regulatory component RAPTOR, leading to dissociation and inhibition of the TOR complex [51]. Thus, there exists a phosphorylation-dependent regulatory loop between ABA core signaling and the TOR complex [51]. In the absence of ABA, SnRK2s and PP2Cs are part of repressor complexes that sequester SnRK1 and prevent it from interacting with and inhibiting TOR. However, in the presence of ABA, these repressor complexes disassemble and release SnRK1, which consequently causes TOR repression [187]. YAK1 (Yet Another Kinase 1), which belongs to the dual specificity tyrosine phosphorylation-regulated kinase family, has recently been demonstrated to be involved in this TOR-ABA signaling. AtYAK1 interacts with RAPTOR and is phosphorylated by TOR. YAK1 mutation suppressed the ABA hypersensitivity of *lst8-1-1* mutants, suggesting that TOR may act through YAK1 to repress ABA signaling [188].

A connection between brassinosteroids (BR) and TOR signaling has been demonstrated, although BR appears to function downstream of TOR. TOR is activated by sugar, leading to inhibition of autophagy, which in turn controls the accumulation of the transcription factor BZR1 (Brassinazole- resistant 1) [22]. In addition, the BR signaling component BIN2 (brassinosteroid insensitive 2) is directly phosphorylated by the TOR kinase substrate S6K2 to regulate photoautotrophic growth in *Arabidopsis* [189]. This suggests that BIN2 can mediate TOR signals to control plant growth. This observation that TOR can promote BR signaling by inhibiting selective autophagic degradation of BZR1 and can inhibit BR signaling by phosphorylating BIN2 probably again indicates a complex regulation in which autophagy can be activated without repressing TOR activity. However, further experimental verification of the link between BR and TOR is still needed, and whether BR can regulate TOR activity is unknown. Other phytohormones including jasmonic acid (JA), salicylic acid (SA), and cytokinin [155,169,171,190] have all been shown to be involved in TOR signaling, but their role is still unclear.

### 4.3. Nutrients

In addition to sugars, nutrients such as sulfur (S), nitrogen (N), and phosphorus (P) are important for plant growth and development, and TOR is one of the pathways through which they signal [2,191,192,193]. The molecular and cellular mechanisms that underlie how TOR transduces, co-ordinates, and integrates multiple nutrient signals are only just emerging.

#### 4.3.1. Sulfur

Sulfur is a key nutrient that is needed for plant growth. It is assimilated into plants by ATP sulfurylase (ATPS) and APS reductase (APR), which reduce sulfate to sulfite, and sulfite reductase (SIR), which further reduces sulfite to sulfide [194]. Analysis of sulfite reductase mutants (*sir1-1*), which have decreased S-precursor supply for cysteine biosynthesis, and WT seedlings grown in sulfate deprivation conditions revealed that S deficiency leads to TOR inhibition, which is coupled with reduced glucose content [191]. Exogenous addition of glucose or grafting of wild-type shoots onto *sir1-1* roots restores TOR activity in the *sir1-1* mutant [191], showing that sulfur likely acts through glucose or energy signaling to influence TOR signaling [191]. The exact mechanism by which sulfur deficiency inhibits TOR activity is not yet known, but one possibility is that sulfur deficiency causes low glucose levels that are sensed by SnRK1, which phosphorylates RAPTOR1B, thus inhibiting TOR activity. Alternatively, TOR activity could be directly controlled by sulfide through an unknown mechanism. More studies are needed to understand how S starvation leads to low glucose content in plants. S deficiency also leads to autophagy induction, which is most likely triggered by decreased TOR activity [191].

#### 4.3.2. Nitrogen

Nitrogen is a very important nutrient and promotes shoot and root growth in *Arabidopsis* [195]. TOR activity is decreased upon N starvation, and resupplying nitrate or ammonium rapidly activates TOR, as measured by S6K phosphorylation by TOR [46]. Additionally, TOR-overexpressing *Arabidopsis* seedlings show increased primary root elongation compared to their WT counterparts when grown in the presence of excess nitrate [148], showing the involvement of TOR signaling in plant N responses. N starvation induces autophagy in photosynthetic eukaryotes [39,75,196,197] and this is probably due to TOR inhibition. In *Chlamydomonas*, phosphoproteomic analysis during N starvation showed a decrease in phosphorylation of the TOR substrate EIF4B which was restored by N re-addition [198]. This indicates that TOR is inactivated by N starvation in *Chlamydomonas* as well. By contrast, increased phosphorylation of other potential TOR substrates was observed, probably due to the activation of alternative kinases including SnRK1 [198]. Surprisingly, growing *Chlamydomonas* under N starvation in different metabolic states revealed that both TOR activity and autophagy are unaffected by N starvation in the absence of a carbon source [199]. Conversely, TOR activity is decreased, and autophagy is upregulated, upon N starvation in the presence of a carbon source that is either photosynthetically fixed or supplied in the growth medium. This effect was determined to be due to disruption of the C/N ratio, in which a high C/N ratio causes an increase in ROS that negatively regulate TOR and cause upregulation of autophagy [199]. It is not clear whether this same molecular mechanism is also present in plants. However, in *Arabidopsis* seedlings, N starvation is associated with accumulation of sugars [200], which could also alter the C/N ratio and cause production of ROS, inhibition of TOR, and induction of autophagy. Whether N starvation-induced sugar accumulation has an effect on TOR signaling as seen for sulfur deficiency, which causes low glucose and TOR inhibition, is not yet known.

#### 4.3.3. Phosphorus

Phosphorus is critical for plant development, and whereas recent studies have elaborated its sensing and signaling mechanisms [193,201,202], it is still unclear if phosphorus regulates TOR signaling in plants. In *Chlamydomonas*, P availability activates TOR, as shown by increased RPS6 (Ribosomal protein small subunit6) phosphorylation [203]. P starvation causes destabilization of LST8 protein and downregulation of TOR activity. P starvation also induces autophagy [203]; however, as this occurs subsequent to the destabilization of LST8, autophagy cannot be responsible for this degradation [203].

*Chlamydomonas* mutants with low levels of inositol phosphates (InsPs) are hypersensitive to rapamycin and other TOR inhibitors. This suggests the involvement of these signaling phosphates in TOR signaling; however, it is not clear whether InsPs work upstream, downstream, or in a parallel pathway to TOR. Treatment of WT *Chlamydomonas* with rapamycin leads to a reduction in InsPs, suggesting that InsPs may function downstream of TOR [204]. An *Arabidopsis* null mutant in inositol pentakisphosphate 2-kinase1 (IPK1) is lethal, while incomplete loss of function *ipk1* mutants have defects in shoots and roots [205]. It is likely that inositol polyphosphates act in phosphorus sensing and TOR signaling in both *Arabidopsis* and *Chlamydomonas*, although the mechanism of action remains to be determined.

### 4.4. Amino Acids

As previously reported in yeast and mammalian cells, the importance of amino acids as activators of the TOR pathway has recently been shown in plants [206,207,208]. Defects in branched chain amino acid (BCAA) biosynthesis lead to reduced TOR kinase activity, as measured by a reduction in S6K phosphorylation [206,207]. Chemical inhibition of BCAA biosynthesis using tribenuron-methyl herbicide (TM) also led to inactivation of TOR and an upregulation of autophagy in *Arabidopsis* seedlings [209]. This indicates that TM-induced BCAA starvation likely inhibits TOR and consequently activates autophagy. BCAAs therefore function as upstream TOR regulators, but the underlying mechanism by which amino acid signals are transduced to TOR is not yet known. Amino acid sensing upstream of TOR in plants is probably carried out by new components given that those described in animals and fungi are absent in plants [210]. Alternatively, could TOR or RAPTOR harbor specific amino acid binding sites that facilitate cell context-dependent autophagy regulation by TORC1? Methods such as homology modeling and reverse genetics approaches may facilitate the discovery of novel amino acid sensors related to those of mammals, while mutant screens may facilitate the study of amino acid interactions, at least with RAPTOR.

### 4.5. Biotic and Abiotic Stress

Biotic stress signals from bacteria, viruses and fungi have in some cases been shown to activate TOR in plants, and TOR-deficient plants are more resistant to some pathogens [190,211,212,213]. When plants are faced with cauliflower mosaic virus infection, the viral transactivating/viroplasmin (TAV) protein directly interacts with and activates TOR. Inhibition of TOR inhibits viral replication for some potyviruses [214] and tombusviruses [215]. Not all potyviruses seem to be affected by TOR signaling. For example, watermelon mosaic virus infection and replication is inhibited by the TOR inhibitor AZD8055 and by silencing of *TOR,* but turnip mosaic virus infection is not [214]. This may be because these two viruses are phylogenetically distinct among potyviruses, and different potyviruses may have differences in their cellular dynamics of infection [214]. TOR inhibition also boosted plant resistance to bacterial pathogens [190,216]. In rice, TOR inhibition caused transcriptional reprogramming by activating defense-related genes and transcription factors such as WRKY and MYB, and by promoting the action of the defense hormones SA and JA [190]. Another possibility to explain the mechanism underlying pathogen resistance upon TOR inhibition could be that TOR inhibition induces autophagy, which has been reported to play a role in plant immune responses [197].

TOR signaling has also been implicated in abiotic stress responses. Most abiotic stresses decrease overall cellular energy levels of plants, which leads to activation of SnRK1, which, in turn, negatively regulates TOR activity [217]. Ectopic expression of the *Arabidopsis* TOR gene in rice and TOR over-expression in *Arabidopsis* [218,219] demonstrated that TOR is required for tolerance of some abiotic stresses. TOR activity is reduced early after the imposition of cold stress [220]. Since inhibition of translation is essential for cold tolerance, and TOR is a positive regulator of translation, the reduced TOR activity upon cold treatment may lead to decreased translation and prepare plants for unfavorable cold conditions [220,221]. Moreover, *TOR* RNAi lines and mutants of *AtTHADA* (an ortholog of the human cold response regulator *HsTHADA*) have decreased TOR activity and are hypersensitive to cold stress, confirming a role for TOR in cold tolerance in plants [221]. The molecular mechanism that controls TOR during cold stress is still unknown.

## 5. TOR Regulation of Autophagy

### 5.1. TOR Is a Negative Regulator of Autophagy

TOR was first reported to be a negative regulator of autophagy in yeast and animals [95,222], and this role is conserved in photosynthetic eukaryotes [75,223]. Inhibition of TOR activity or down-regulation of *TOR* by RNAi in *Arabidopsis* results in activation of autophagy, with an increase in the formation of autophagosomes and an upregulation of some *ATG* genes [19,223]. The constitutive autophagy in *TOR* RNAi lines was dependent on the core autophagy gene *ATG18a,* indicating that TOR regulates the canonical autophagy pathway [223]. Inhibition of TOR by rapamycin [75] and in TOR insertional mutants [199] in *Chlamydomonas* caused downregulation of TOR activity and upregulation of autophagy [75], showing that negative regulation of autophagy by TOR is conserved in *Chlamydomonas*.

As in land plants, starvation for nitrogen and carbon, known TOR upstream signals, resulted in upregulation of autophagy [75] in *Chlamydomonas*. Thus, TOR likely functions in the control of autophagy in response to nutrient deficiency and this function is conserved in both land plants and algae. Other TOR upstream signals such as sulfur [191], amino acids [209], and biotic and abiotic stress have all been implicated in regulation of autophagy in plants. Purine nucleotide depletion also activates autophagy in *Arabidopsis* via inhibition of TOR [224]. Overexpression of TOR and exogenous addition of the auxin NAA, an upstream activator of TOR, prevented autophagy activation in response to several abiotic stresses [19], confirming the importance of TOR in autophagy regulation in plants. Taken together, these studies show that TOR negatively regulates autophagy in photosynthetic eukaryotes as it does in other organisms.

**Figure 4:**
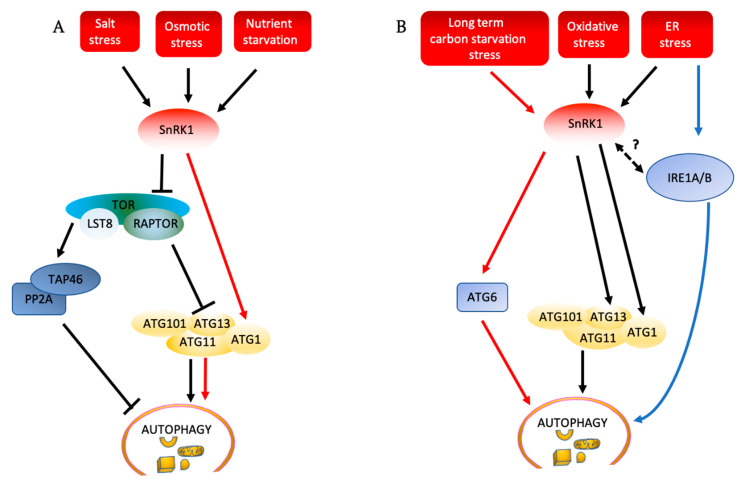
Autophagy induction pathways in plants (**A**) TOR must be repressed for salt stress, osmotic stress, and nutrient deficiency to activate autophagy. These stresses activate SnRK1, which inhibits the TOR complex, and activate the ATG1 complex. SnRK1 can also directly phosphorylate and activate the ATG1 complex, leading to activation of autophagy (red arrows). Inhibition of TOR by SnRK1 can lead to inactivation of TAP46, a regulatory subunit of PP2A, leading to activation of autophagy. (**B**) SnRK1 phosphorylates ATG6 to activate autophagy in response to long-term carbon starvation, independently of TOR (red arrows). ER stress and oxidative stress induce autophagy through SnRK1 and ATG1, independently of TOR. ER stress also induces autophagy through IRE1, although the relationship to SnRK1 is unknown. Solid arrows show pathways that have been experimentally demonstrated, while dashed arrows show potential pathways that still need to be confirmed. ATG: autophagy-related; IRE1A/B: inositol-requiring enzyme1/2; LST8: lethal with sec thirteen 8; PP2A: protein phosphatase 2A; RAPTOR: regulatory associated protein of TOR; SnRK1: SNF1-related protein kinase-1; TAP46: protein phosphatase 2A-associated protein; TOR: target of rapamycin.

In *Arabidopsis*, the relationship between TOR activity and autophagy depends on the type of stress to which plants are exposed. In some stresses, such as nutrient deficiency, salt, and osmotic stress, TOR activity must be repressed for autophagy to be activated, while in others, for example oxidative stress or endoplasmic reticulum stress, autophagy can be activated even in the presence of active TOR [19,83] (Figure 4A,B). It is not clear why activation of autophagy in some stresses proceeds only after TOR inactivation while in some others it proceeds regardless of TOR activity status. One possible explanation could be the physiological differences triggered by the different stresses. Many environmental stresses are known to cause accumulation of ROS in plant cells that act as upstream signals for autophagy induction. For example, autophagy induction upon nutrient starvation, exposure to salt stress or hypoxia [225] is dependent on upstream ROS signals generated by the plasma membrane nicotinamide adenine dinucleotide phosphate (NADPH) oxidase, whereas inhibition of NADPH oxidase had no effect on autophagy when induced by osmotic stress. This discrepancy shows that the autophagy in nutrient starvation and salt stress is modulated in a ROS-dependent manner, requiring the signals generated by NADPH oxidase, which is well-characterized as triggering a set of stress responses [226]. By contrast, autophagy is induced by osmotic stress in a ROS independent manner, or potentially osmotic stress leads to the production of other signals besides ROS that continue to induce autophagy in the presence of ROS production inhibitors. In mice, it was demonstrated that ROS led to TOR inactivation and activation of autophagy [227]. Based on this, one possibility is that ROS inactivates TOR and induces autophagy in photosynthetic organisms as well. The inhibition of NADPH oxidase in TOR-RNAi lines [223], which have decreased TOR expression and activity, has no effect on autophagy, suggesting that alternatively, TOR and ROS may act independently in signaling to the autophagy machinery in response to environmental cues. It is tempting to speculate that different stresses may produce different signals which might then activate autophagy via TOR signaling or via TOR independent pathways, with the complexity of signaling inputs mirroring the diversity of autophagy functions and responses. Different stresses could also produce more than one signal, and distinct combinations of signals could produce different outcomes, explaining the seemingly contradictory results under different conditions.

### 5.2. Mechanism of Regulation of Autophagy by TOR

As in other organisms, the TOR complex in plants exerts its influence on autophagy through its regulation of the ATG1-ATG13 complex in response to nutrient availability. In plants, the ATG1/ATG13 complex is conserved and responds to nutrient status. *Arabidopsis* has four ATG1 isoforms, ATG1a, ATG1b, ATG1c, and ATG1t, and two ATG13 isoforms, ATG13a and ATG13b, with these multiple copies probably functionally redundant [228,229]. Both ATG1 and ATG13 have been identified as phosphoproteins that are reversibly phosphorylated by TOR [230]. ATG1a is hyper-phosphorylated, probably by the SnRK1 complex, while ATG13a is hypo-phosphorylated during nutrient starvation [228]. As in yeast and animals, *Arabidopsis* ATG13 proteins contain a canonical TOS (TOR signaling) motif, which mediates binding to RAPTOR. Deletion of this five amino acid core sequence element (FSDIF) was accompanied by increased autophagy and reduction in ATG13 phosphorylation levels. This shows that ATG13 phosphorylation by TOR is mediated by the interaction of RAPTOR with ATG13 through the TOS core element and is important for autophagy regulation [231]. The ATG1/ATG13 complex therefore regulates autophagy in plants in response to nutrient status, probably via TOR kinase activity [228].

SnRK1 has also been implicated as an activator of autophagy, acting both upstream of and independently of TOR. Overexpression of the *Arabidopsis* SnRK1 catalytic subunit KIN10 increased autophagy [232,233], and KIN10 interacts with and can phosphorylate RAPTOR1B [166]. This suggests that, as seen in mammals and yeast [52,92], SnRK1 inactivates TOR by phosphorylation of RAPTOR1B, thus activating autophagy in response to stress. Knockout of the *KIN10* gene disrupted abiotic stress-induced autophagy, but not the constitutive autophagy caused by inhibition of TOR [233], showing that TOR acts downstream of KIN10 to regulate autophagy under conditions in which activation of autophagy depends on repression of TOR activity.

As discussed above, TAP46 is a regulatory subunit of PP2A and a phosphorylation target of TOR. Over- or under-expression of TAP46 results in an increase or decrease in TOR activity, respectively, suggesting that TAP46 positively regulates TOR activity [234]. Virus-induced silencing of *TAP46* resulted in activation of autophagy in tobacco (*Nicotiana tabacum*) and *Arabidopsis* protoplasts [235]. TAP46 therefore negatively regulates autophagy by increasing TOR activity (Figure 4A).

Mutation of *KIN10* prevented activation of autophagy by oxidative or ER stress, yet over-expression of TOR did not [233,236]. Oxidative stress and ER stress therefore activate autophagy via a TOR-independent but SnRK1-dependent pathway (Figure 4B). This is further illustrated by phosphate starvation-induced autophagy, which was also found to be ER stress dependent. This autophagy was not blocked by TOR overexpression, further confirming ER stress-induced autophagy as TOR independent. Pi starvation led to apoplastic ROS generation, suggesting that ROS were likely acting as the signal for ER stress response activation and/or that ROS were the signal for autophagy induction via a TOR-independent pathway. The autophagy induced by Pi-starvation was localized to root tips, as opposed to the systemic autophagy induced by other stresses. It is therefore possible that signals that induce systemic autophagy act through TORC1, while local responses do not require TORC1-regulation, although this requires more experimental evidence. As overexpression of KIN10 increased ATG1 phosphorylation levels, and KIN10 interacted with ATG1a and ATG13a in vitro, KIN10 most likely acts by direct phosphorylation of ATG1 [232] in TOR-independent autophagy. Recently, it has been shown that SnRK1 also directly phosphorylates ATG6, a component of the PI3K (phosphoinositide 3-kinase) complex, to activate autophagy in response to long term carbon starvation (Figure 4B) [229]. It is still not clear why autophagy is regulated through different pathways dependent on the environmental conditions. Presumably, in the context of maintaining homeostasis and ensuring cell survival, in specific environments, both the magnitude, as well as the timing and duration of autophagy induction are controlled to suit the cellular demand. For instance, hypothetically, a short period of amino acid starvation may have limited physiological consequences compared to hypoxia and, therefore, the extent of autophagy activation during hypoxia is enormous and may require extra mechanisms to avert detrimental consequences. Some stress signals, as well as autophagy responses, have also been shown to be systemic in nature, while others are locally coordinated. It will be necessary to evaluate the potential triggers for this spatial signal response, and the role of TOR if any in this context.

## 6. Perspectives

The TOR complex is evolutionarily conserved across eukaryotes and is regulated by upstream factors, including hormones, biotic and abiotic stresses, amino acids, nutrients, and energy. These upstream signals affect the ability of TOR to interact with and phosphorylate downstream effectors to promote ribosome biogenesis, translation and transcription, and to repress autophagy when conditions are favorable. The identification of chemical inhibitors of TOR, use of inducible *TOR* silencing lines and mutants defective in the TORC1 components RAPTOR and LST8, coupled with the establishment of reliable biochemical assays for TOR activity by measurement of S6K phosphorylation levels, have allowed progress in understanding the TOR pathway in plants.

Recent findings have demonstrated that TOR is not the sole regulator of autophagy in plants. Multiple pathways, some requiring repression of TOR activity and some not, depending on the type of stress, have been established. Despite the progress made in understanding the role of TOR in autophagy regulation, research is still needed to identify additional autophagy regulators and TOR upstream signals. The stress sensors that signal to SnRK1 and TOR to regulate autophagy are largely still unknown. It is also unknown whether some stresses induce autophagy independently of both TOR and SnRK1. Analysis of substrates of TOR and SnRK1 [166,230] presents an opportunity for the discovery of other proteins that may be involved in autophagy in plants.

Another constraint in plant TOR signaling research stems from lack of the known structure of the TOR kinase complex and its subunits, coupled with the embryo lethality of loss-of function *tor* mutants. As many of the known factors that regulate TOR in animals are absent from plants, it is likely that plants contain as yet unknown interacting partners or regulators of TOR activity. In order to predict new interaction domains or residues in TOR, as well as regulatory sites, it will be necessary to use biophysical or computational tools, such as in silico structural modeling approaches, which may facilitate TORC1-substrate complex elucidation and context-specific functional determination. Moreover, in combination with biochemical methods, such approaches may facilitate discovery of unknown TOR regulators as well as substrates in the TOR pathway that regulate autophagy. For instance, questions that could be asked in this regard are; how is the TOR complex assembled in response to specific stimuli or cellular conditions? Is the TORC1 basic structure influenced by specific substrates? How does TOR achieve regulation of autophagy in specific cell types or tissues? Are TOR-autophagy signaling cascades transient in nature, and if so, for how long do they last and in which plant tissues or conditions are these transient interactions favored?

Although trying to understand TOR signaling with respect to a single stimulus has been the order of the day [237,238,239], it goes without saying that in the real outside environment, plants experience multiple stimuli, which may not be achieved in the laboratory setting in which most experiments are done. For instance, plants usually face a combination of saline, extreme heat or reduced water availability conditions, but how these external stimuli are sensed and integrated as a set is understudied. Moreover, it is probable that TOR, a master signal integrator, contributes significantly to plant survival when faced with multiple stresses, although more experimental evidence is needed [239]. For instance, consistent with stress-specific tissue- or cell-specific plant responses, TORC1 may mediate responses specific to a combination of stresses or stimuli to ensure acclimation [239,240,241] by regulating processes such as autophagy in a context-specific manner. Whether this regulation is achieved post transcriptionally or at a protein level will be an interesting question to pursue further.

## Abbreviations

ABA	Abscisic acid
AMBRA1	Autophagy/beclin-1 regulator 1
AMPK	Adenosine monophosphate-activated protein kinase
APR	APS reductase
Arg	Arginine
ATG	Autophagy-related
ATPS	ATP sulfurylase
BCAA	Branched chain amino acid
BIN2	Brassinosteroid insensitive 2
BR	Brassinosteroid
BZR1	Brassinazole- resistant 1
C/N	Carbon/nitrogen
DAPK2	Death-associated protein kinase 2
DEPTOR	DEP-domain-containing mTOR-interacting protein
EIF4B	Eukaryotic translation initiation factor 4B
FAT	Focal adhesion target
FATC	FAT-carboxy terminal domain
FRB	FKBP12-rapamycin binding domain
FIP200	Focal adhesion kinase family interacting protein of 200 kDa
GATOR2	GAP activity towards Rags 2
Glc	Glucose
HAC1	Histone acetyltransferase 1
HEAT	Huntingtin-Elongation factor 3-regulatory subunit A of PP2A-TOR1
HLP1	Hikeshi-like Protein 1
InsPs	Inositol phosphates
IPK1	Inositol pentakisphosphate 2-kinase1
IRE1A/B	Inositol-requiring enzyme1/2
JA	Jasmonic acid
KIN10	Kinase homolog 10
KOG1	Kontroller of growth 1
Leu	Leucine
LST8	Lethal with sec thirteen 8
N	Nitrogen
NAA	Naphthaleneacetic acid
NADPH	Nicotinamide adenine dinucleotide phosphate
P	Phosphorus
PP2C	Protein phosphatase 2C
PRAS40	40 kDa pro-rich AKT substrate
PYL	Pyrabactin resistance 1 like
RAG	Ras-related GTP binding protein
RAPTOR	Regulatory-associated protein of TOR
RHEB	Ras homolog enriched in brain
RNC	RAPTOR N-terminus conserved domain
ROP	Rho-like GTPases
ROS	Reactive oxygen species
RPS6	Ribosomal protein small subunit6
RUVBL	RuvB Like AAA ATPase
S	Sulfur
SA	Salicylic acid
SIR	Sulfite reductase
SLC38A9	Solute carrier family 38
SnRK1/2	SNF1-related protein kinase-1/2
S6K	S 6 kinase
TAP46	Protein phosphatase 2A-associated protein
TAV	Viral transactivating/viroplasmin
TE	Tracheary elements
Tco89p	89 kDa protein of TOR complex one
TFEB	Transcription factor EB
TLR4	Toll-like receptor 4
TOR	Target of rapamycin
TORC1	Target of rapamycin complex 1
TSC	Tuberous sclerosis complex
TTT	Tel2-Tti1-Tti2
ULK1	UNC-51-like kinase 1
V-ATPase	Vacuolar-type H^+^-ATPase
WAC	WW domain containing adaptor with coiled coil
WD40	Tryptophan-aspartic acid repeats of 40 amino acids
YAK1	Yet another kinase 1
